# Elevated plasma D-dimer levels are associated with short-term poor outcome in patients with acute ischemic stroke: a prospective, observational study

**DOI:** 10.1186/s12883-019-1386-3

**Published:** 2019-07-22

**Authors:** Tao Yao, Bo-Lin Tian, Gang Li, Qin Cui, Cui-fang Wang, Qi Zhang, Bo Peng, Yan Gao, Yan-Qiang Zhan, Dan Hu, Lu Xu, Gao-Hua Wang

**Affiliations:** 1 Department of Neurology, Renmin Hospital of Wuhan University, Wuhan, 430060 China; 2 Department of Neurology, Dawu County Hospital of Traditional Chinese Medicine, Hubei, 432800 China; 3 Emergency Department, Hubei Provincial Hospital of Traditional Chinese Medicine, Wuhan, 430061 China; 4 Department of Psychiatry, Renmin Hospital of Wuhan University, Wuhan, 430060 China; 5 Institute of Neuropsychiatry, Renmin Hospital of Wuhan University, Wuhan, 430060 China

**Keywords:** Acute ischemic stroke, D-dimer, Outcome, Modified Rankin scale

## Abstract

**Background:**

Elevated levels of plasma D-dimer increase the risk of ischemic stroke, stroke severity, and the progression of stroke status, but the association between plasma D-dimer level and functional outcome is unclear. The aim of this study is to investigate whether plasma D-dimer level is a determinant of short-term poor functional outcome in patients with acute ischemic stroke (AIS).

**Methods:**

This prospective study included 877 Chinese patients with AIS admitted to Renmin Hospital of Wuhan University within 72 h of symptom onset. Patients were categorized by plasma D-dimer level: Quartile 1(≤0.24 mg/L), Quartile 2 (0.25–0.56 mg/L), Quartile 3 (0.57–1.78 mg/L), and Quartile 4 (> 1.78 mg/L). The medical record of each patient was reviewed, and demographic, clinical, laboratory and neuroimaging information was abstracted. Functional outcome at 90 days was assessed with the modified Rankin Scale.

**Results:**

Poor outcome was present in 302 (34.4%) of the 877 patients that were included in the study (mean age, 64 years; male, 68.5%). After adjustment for potential confounding variables, higher plasma D-dimer level on admission was associated with poor outcome (adjusted odds ratio 2.257, 95% confidence interval 1.349–3.777 for Q4:Q1; P trend = 0.004). According to receiver operating characteristic (ROC) analysis, the best discriminating factor for poor outcome was a plasma D-dimer level ≥ 0.315 mg/L (area under the ROC curve 0.657; sensitivity 83.8%; specificity 41.4%).

**Conclusion:**

Elevated plasma D-dimer levels on admission are significantly associated with poor outcome after admission for AIS, suggesting the potential role of plasma D-dimer level as a predictive marker for short-term poor outcome in patients with AIS.

## Background

Epidemiological investigations have concluded that stroke is a leading cause of adult disability and mortality, and poses a serious public health burden worldwide [1–3]. Recently, the multicenter Global Burden of Disease (GBD 2016) Study found that the risk of ischemic stroke was 18.3% and the risk of hemorrhagic stroke was 8.2% among adults 25 years of age or older [4]. As a predominant stroke subtype in Chinese populations [5], acute ischemic stroke (AIS) reached 66.4% among the stroke subtypes between September 2007 and August 2008 in the Chinese National Stroke Registry [6]. Because of the high morbidity and risk of disability after AIS, an estimation of prognosis is an emergent issue, especially when physicians are confronted with concerns from patients and families. Recent studies have assessed prognostic factors such as glycemic index, body mass index (BMI), and uric acid, but their prognostic values in relation to AIS was inconsistent [7–12]. For specific management of stroke rehabilitation in regard to the neurological functional outcome, identifying more powerful predictors of clinical prognosis is warranted.

D-dimer is a soluble fibrin degradation final product and derived from the cross-linked fibrin network as it undergoes plasmin-mediated degradation. The plasma D-dimer level increases during blood thrombosis and degradation of fibrin, therefore plasma D-dimer could be a biological marker of hemostatic abnormalities and thrombosis [13]. Elevated plasma D-dimer levels are reportedly a determinant of stroke progression [14], infarction volume [15], and the incidence of stroke [16]. Recently, many studies have investigated whether plasma D-dimer levels are a determinant of poor functional outcomes after AIS, however, the conclusions of the studies were controversial [17–20]. Some investigators have found that plasma D-dimer levels could independently predict poor functional outcomes in patients with AIS [17, 18], while other investigators have reported conflicting results [19, 20].

Accordingly, the aim of this study was to investigate whether elevated plasma D-dimer levels could be a significant determinant of poor outcome after admission for AIS.

## Methods

### Study population

This was a prospective follow-up study. Data were retrospectively analyzed from a prospective registry. We enrolled 877 consecutive Chinese patients with AIS at Renmin Hospital of Wuhan University from January 2017 to August 2018. All patients were admitted within 72 h of experiencing a new focal or global neurological event. AIS was diagnosed according to the World Health Organization criteria [21] combined with brain computed tomography or magnetic resonance confirmation within 72 h. Patients were excluded if any of the following criteria were met: a delay of 72 h from symptom recognition to admission, age younger than 18 years, preexisting significant disability (defined as modified Rankin scale, mRS ≥ 2) from any condition, intracranial hemorrhage, malignancy, febrile disorders, and acute or chronic inflammatory disease at study enrollment. Each participant was followed up after 3 months via telephone, email, and face to face. The study protocol complied with the Declaration of Helsinki and was approved by the Wuhan University Ethics Committee.

### Demographic and clinical assessment

Socio-demographic, self-reported medical history, and vascular risk biomarker data were assessed and included: age, sex, BMI, history of hypertension, diabetes, alcohol consumption, smoking, dyslipidemia, atrial fibrillation, previous stroke, and coronary artery disease (CAD). The National Institutes of Health Stroke Scale (NIHSS) scores were used by stroke neurologists to assess neurological deficit when the patients were admitted [22]. Stroke subtype was classified according to the Trial of Org 10172 in acute stroke treatment (TOAST classification) criteria [23], which distinguished large-artery arteriosclerosis, small-artery occlusion, cardioembolism, other causative factor, and undetermined causative factor.

Fasting plasma glucose (FPG) and plasma D-dimer level were measured in the morning after at least 8 h of fasting. Plasma D-dimer level was measured for all patients with a particle-enhanced immunoturbidimetric assay in a calibrated SYSMEX7000 analyzer (Sysmex Corporation, Hyogo, Japan). The normal range of morning plasma D-dimer concentration in our hospital laboratory is 0–0.55 mg/L.

### Follow-up and short-term outcomes

Patient follow-up was performed at 90 days after stroke onset. The prognosis outcome was assessed with modified Rankin Scale (mRS) via telephone, email, and face to face by a trained research nurse or neurologist. A good functional outcome was defined as an mRS of 0–2 points, whereas a poor outcome was defined as an mRS of 3–6 points.

### Statistical analysis

For continuous variables, data are expressed either as the means ± standard deviations (SD) or medians (interquartile ranges, IQR). Categorical variables are expressed as frequencies and percentages. The patients were categorized into two groups according to prognosis outcome (good outcome vs poor outcome). A two-group comparison of normally distributed continuous variables was assessed using independent t-tests. The non-parametric Mann–Whitney U test was used for continuous variables that were not normally distributed. The χ^2^ test was used for categorical variables. Furthermore, we categorized the patients into four quartile groups according to their plasma D-dimer level at admission. A four-group comparison was assessed using the χ^2^ test, one-way analysis of variance (ANOVA) and Mann–Whitney U tests, as appropriate. Multivariate analysis adjustment for variables was performed for the correlation between the quartiles of plasma D-dimer levels and poor outcome by logistic regression analysis, which used methods from previous studies [24, 25]. Results were expressed as adjusted odds ratios (OR) with the corresponding 95% confidence intervals (CIs). Receiver operating characteristic (ROC) curves were utilized to evaluate the accuracy of plasma D-dimer level to predict AIS poor neurological outcome. The area under the curve (AUC) was calculated as a measurement of the accuracy of the test. All statistical analysis was performed with SPSS for Windows, version 22.0 (SPSS Inc., Chicago, IL, USA). *P* < 0.05 was considered statistically significant.

## Results

### Baseline characteristics of the patients

**Fig. 1:**
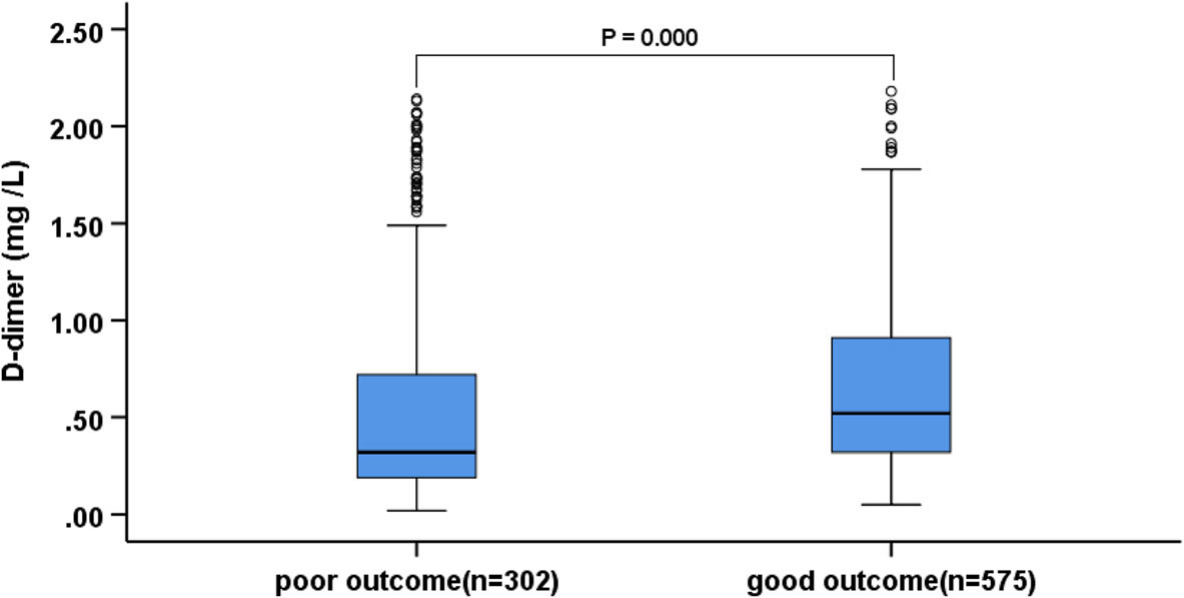
Plasma D-dimer level in patients with good or poor outcomes. Mann–Whitney U-test (Z = −7.655, *P* = 0.000)

A total of 877 AIS patients (median age 64 years, 68.5% male) who met the inclusion criteria were recruited for this study. The variables associated with functional outcome of AIS included sex, age, BMI, vascular risk factors (smoking, alcohol drinking, atrial fibrillation, diabetes, hypertension, CAD, dyslipidemia, previous stroke), baseline systolic blood pressure (SBP), baseline systolic-diastolic blood pressure (DBP), FBG, baseline NIHSS scores and stroke subtype. The median plasma D-dimer level on admission was 0.56 (0.24–1.79) mg /L, and the median NIHSS score on admission was 5 (3–8). In this study, 575 patients (65.6%) presented with good outcomes, 302 patients (34.4%) presented with poor outcomes, and 77 patients (8.8%) died among the 877 patients within 90 days. The baseline characteristics and outcome of the patients with AIS are described in Table 1. The sex, age, BMI, smoker, history of atrial fibrillation, FBG, plasma D-dimer level, baseline NIHSS scores, and stroke etiology were markedly associated with the outcomes of AIS at 90 days (*P* < 0.05 for all). Figure 1 shows the plasma D-dimer level between two functional outcome groups. In the patients with poor outcome, plasma D-dimer levels were significantly higher compared with those in patients with good outcomes [0.88 (IQR, 0.42–2.72) mg/L vs 0.46 (IQR, 0.21–1.32) mg/L; Z = − 7.655, *P* = 0.000].

**Table 1:** Baseline characteristics of the study patients grouped by 90-day functional outcome

Variable	all	good outcome	poor outcome	*P* value
	(*n* = 877)	(*n* = 575)	(*n* = 302)	
Age (years), median (IQR)	64.00 (54.50–73.00)	62 (52–70)	68 (60–77.25)	0.000
Sex (male), n (%)	601 (68.5)	413 (71.8)	188 (62.3)	0.004
BMI (kg/m2),(Mean ± SD)	25.09 ± 3.64	24.76 ± 3.54	25.70 ± 3.74	0.000
Smoker, n (%)	337 (38.4)	217 (37.7)	120 (39.7)	0.307
Alcohol drinkers, n (%)	192 (21.9)	137 (23.8)	55 (18.2)	0.056
Hypertension, n (%)	531 (60.5)	336 (58.4)	195 (64.6)	0.077
Diabetes mellitus, n (%)	281 (32.0)	172 (29.9)	109 (36.1)	0.062
CAD, n (%)	106 (12.1)	61 (10.6)	45 (14.9)	0.064
Atrial fibrillation, n (%)	110 (12.5)	44 (7.7)	66 (21.9)	0.000
Dyslipidemia, n (%)	309 (35.2)	209 (36.3)	100 (33.1)	0.341
Previous stroke, n (%)	116 (13.2)	73 (12.7)	43 (14.2)	0.522
NIHSS on admission, median (IQR)	5 (3–8)	5 (3–7)	7 (5–8)	0.000
SBP (mmHg), median (IQR)	147 (131–164)	147 (132–162)	147 (130–165)	0.949
DBP (mmHg), median (IQR)	83 (75–92)	83 (75–92)	83 (74–92)	0.229
FBG (mmol/L), median (IQR)	6 (4.81–8.22)	5.62 (4.63–7.50)	6.70 (5.46–9.50)	0.000
D-dimer (mg /L), median (IQR)	0.56 (0.24–1.79)	0.46 (0.21–1.32)	0.88 (0.42_2.72)	0.000
Stroke etiology, n (%)				0.000
Large-vessel occlusive	344 (39.2)	176 (30.6)	168 (55.6)	
Small-vessel occlusive	366 (41.7)	312 (54.3)	54 (17.9)	
Cardioembolic	88 (10)	32 (5.6)	56 (18.5)	
Other	30 (3.4)	21 (3.7)	9 (3.0)	
Undetermined	49 (5.6)	34 (5.9)	15 (5.0)	

*BMI* body mass index, *CAD* coronary artery disease, *NIHSS* National Institutes of Health Stroke Scale, *SBP* systolic blood pressure, *DBP* diastolic blood pressure, *IQR* interquartile range, *SD* standard deviation

^a^ *P* value was assessed using χ^2^ test, independent t-tests, or Mann–Whitney U test, as appropriate

### Correlation between plasma D-dimer level and 90-day functional outcome

**Fig. 2:**
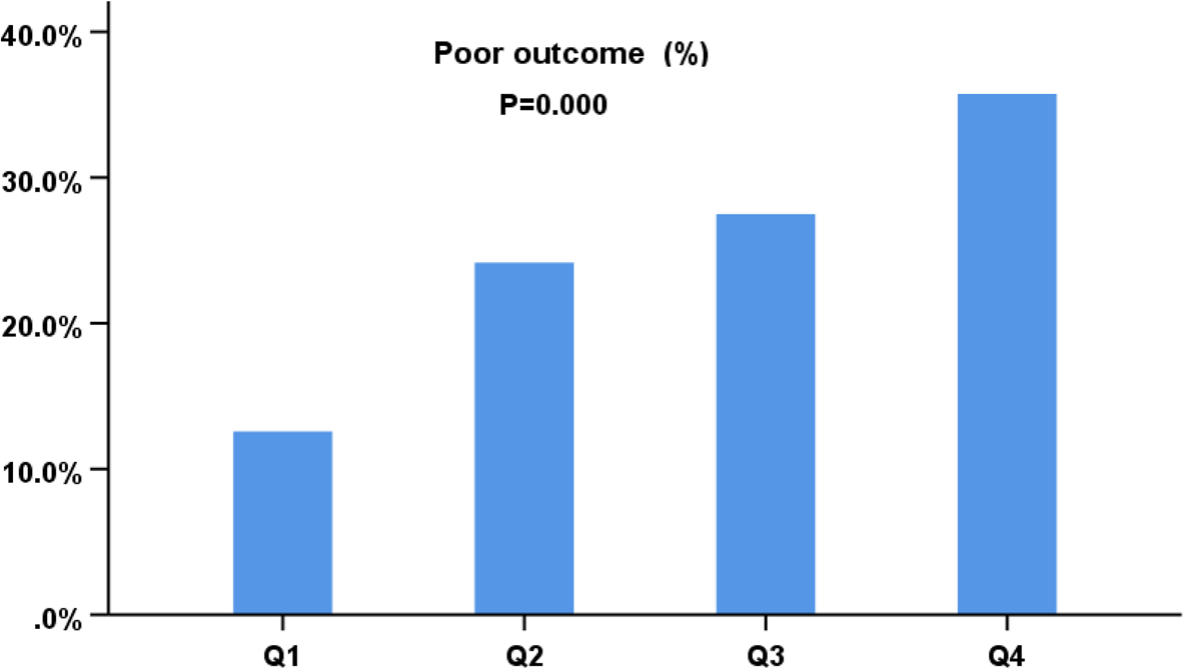
Comparisons of the outcome of AIS patients according to quartiles for plasma D-dimer levels. χ^2^ Test for trend (χ^2^ = 53.724, *P* = 0.000)

Patients were stratified into four groups according to plasma D-dimer quartiles: Plasma D-dimer levels ≤0.24 (*n* = 226), 0.25–0.56 (*n* = 213), 0.57–1.78 (*n* = 219), and > 1.78 mg /L (*n* = 219) (Table 2). Among the four groups, there were no significant differences in the history of hypertension, diabetes, dyslipidemia, previous stroke, drinking alcohol, FBG, and baseline SBP (*P* > 0.05 for all). Age, sex, BMI, smoking, atrial fibrillation, baseline DBP, baseline NIHSS scores, stroke etiology, and mortality differed among the four groups (*P* < 0.05 for all). The unadjusted comparisons of the four groups revealed more poor outcomes among the higher quartiles of Plasma D-dimer levels (χ^2^ = 53.724, *P* = 0.000) (Fig. 2).

**Table 2:** Baseline characteristics of the study patients grouped by plasma D-dimer quartile

	Quartile 1	Quartile 2	Quartile 3	Quartile 4	*P*value^a^
	(≤ 0.24) *n* = 226	(0.25–0.56) *n* = 213	(0.57–1.78) *n* = 219	(>1.78) *n* = 219	
Age (years), median (IQR)	59 (50–66)	66 (58–74)	66 (53–76)	66 (57–76)	0.000
Sex (male), n (%)	172 (76.1)	152 (71.4)	142 (64.8)	135 (61.6)	0.005
BMI (kg/m2),(Mean ± SD)	24.89 ± 3.52	24.79 ± 3.56	24.99 ± 3.64	25.69 ± 3.78	0.040
Smoker, n (%)	102 (45.1)	90 (42.3)	74 (33.8)	71 (32.4)	0.012
Alcohol drinker, n (%)	56 (24.8)	51 (23.9)	44 (20.1)	41 (18.7)	0.344
Hypertension, n (%)	138 (61.1)	131 (63.5)	130 (59.4)	133 (60.7)	0.972
Diabetes mellitus, n (%)	83 (36.7)	71 (33.3)	55 (25.1)	72 (32.9)	0.062
CAD, n (%)	18 (8.0)	24 (11.3)	27 (12.3)	37 (16.9)	0.036
Atrial fibrillation, n (%)	10 (4.4)	23 (10.8)	30 (13.7)	47 (21.5)	0.000
Dyslipidemia, n (%)	90 (39.8)	80 (37.6)	63 (28.8)	76 (34.7)	0.085
Previous stroke, n (%)	22 (9.7)	38 (17.8)	28 (12.8)	28 (12.8)	0.093
NIHSS on admission, median (IQR)	4 (3–7)	5 (3–7)	6 (3–7)	7 (5–10)	0.000
SBP (mmHg), median (IQR)	148 (134–165)	145 (131–162)	144 (130–162)	152 (134–166)	0.221
DBP (mmHg), median (IQR)	85 (78–95)	82 (76–90)	80 (74–90)	84 (76–93)	0.001
FBG (mmol/L), median (IQR)	5.7 (4.66–8.23)	6.01 (4.95–7.85)	5.90 (4.78–7.46)	6.23 (5.00–9.10)	0.089
D-dimer (mg /L), median (IQR)	0.17 (0.12–0.21)	0.38 (0.31–0.48)	0.92 (0.72–1.27)	3.06 (2.45–3.97)	0.000
Stroke etiology, n (%)					0.000
Large-vessel occlusive	81 (35.8)	82 (38.5)	86 (39.3)	95 (43.4)	
Small-vessel occlusive	118 (52.2)	93 (43.7)	91 (41.6)	64 (29.2)	
Cardioembolic	7 (3.1)	18 (8.5)	24 (11)	39 (19.8)	
Other	12 (5.3)	7 (3.3)	5 (2.3)	6 (2.7)	
Unknown	8 (3.5)	13 (6.1)	13 (5.9)	15 (6.8)	
Mortality, n (%)	4 (1.8)	13 (6.1)	24 (11)	36 (16.4)	0.000

*BMI* body mass index, *CAD* coronary artery disease, *NIHSS* National Institutes of Health Stroke Scale, *SBP* systolic blood pressure, *DBP* diastolic blood pressure, *IQR* interquartile range, *SD* standard deviation

^a^ *P* value was assessed χ^2^ test, ANOVA or Mann–Whitney U tests, as appropriate

**Fig. 3:**
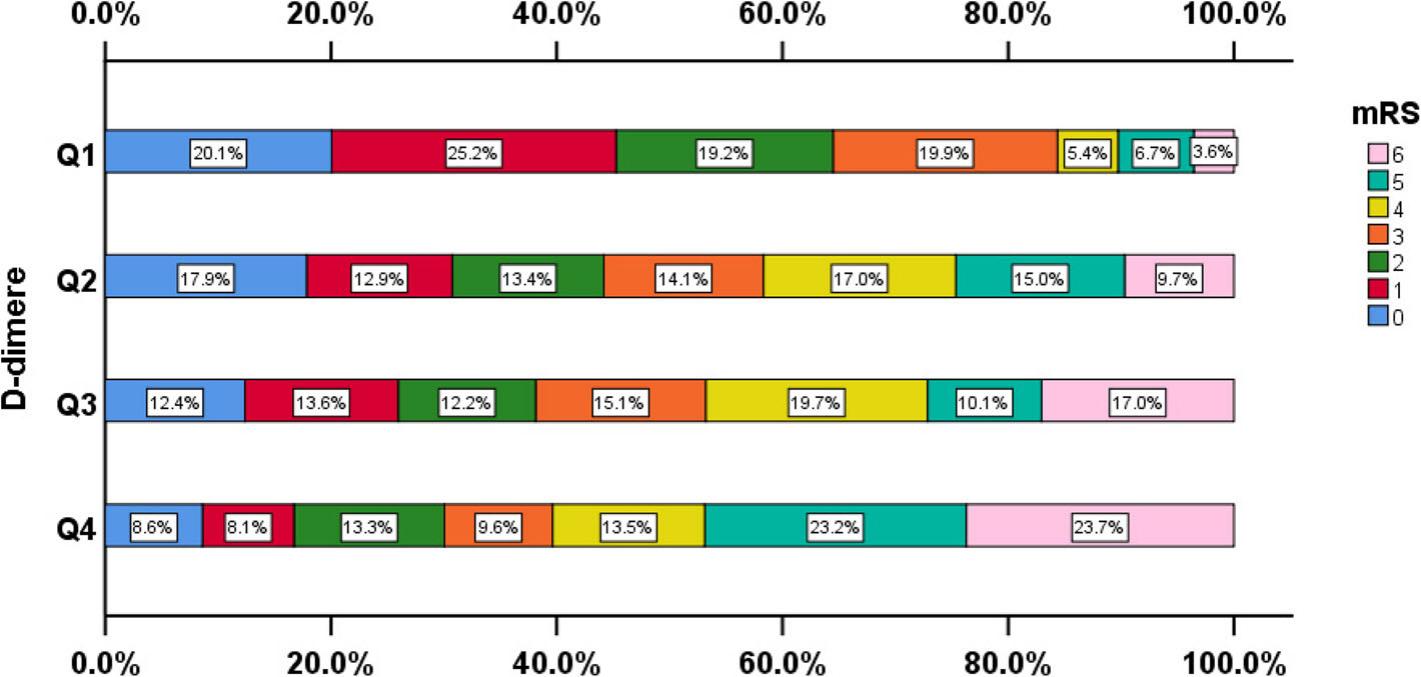
Functional outcome stratified for plasma D-dimer levels. χ ^2^ Test for trend (χ ^2^ = 100.316, *P* = 0.000). mRS: modified Rankin scale

Functional outcome stratified for plasma D-dimer levels is shown in Fig. 3. Univariate analysis shows a clear relationship between admission plasma D-dimer levels and mRS using the χ^2^ Test (χ^2^ = 877.000, P trend = 0.000). Furthermore, the correlation between plasma D-dimer levels and poor outcome after adjustment for variables are detailed in Table 3. In patients with high plasma D-dimer levels, the risk of poor functional outcome at 90 days was significantly increased when compared with the group with low plasma D-dimer levels (P trend = 0.000, OR = 3.800, 95% CI = 2.420–5.965 for Q4: Q1; adjusted for age, sex, and BMI). Additional adjustment for smokers, alcohol drinkers, atrial fibrillation, diabetes, hypertension, CAD, dyslipidemia, previous stroke, baseline SBP, baseline DBP, FBG, baseline NIHSS scores, and stroke etiology did not influence this finding. An overall OR of 2.257 (P trend = 0.004, 95% CI = 1.349–3.777 for Q4: Q1) was found for patients with high plasma D-dimer levels.

**Table 3:** Adjusted odds ratios for poor outcome according to plasma D-dimer levels

	Quartile 1	Quartile 2	*P* value	Quartile 3	*P* value	Quartile 4	*P* value	P for trend
OR (95% CI)^a^	1	2.139 (1.348–3.393)	0.001	2.518 (1.596–3.974)	0.000	3.800 (2.420–5.965)	0.000	0.000
OR (95% CI)^b^	1	2.021 (1.225–3.334)	0.006	2.503 (1.527–4.105)	0.000	3.181 (1.964–5.201)	0.000	0.000
OR (95% CI)^c^	1	2.028 (1.208–3.405)	0.007	2.246 (1.345–3.749)	0.002	2.257 (1.349–3.777)	0.002	0.004

*OR* odds ratio, *CI* confidence interval, *OR*^a^ adjusted for age, sex, and body mass index, *OR*^b^ as note a with additional adjustment for smokers, alcohol drinkers, atrial fibrillation, diabetes, hypertension, CAD, dyslipidemia, previous stroke, and stroke etiology, *OR*^c^ as note b with additional adjustment for baseline SBP, DBP, FBG, and NIHSS, *SBP* systolic blood pressure, *DBP* diastolic blood pressure, *FPG* fasting plasma glucose, *NIHSS* National Institutes of Health Stroke Scale

### Predictive values of plasma D-dimer level in patient outcome

**Fig. 4:**
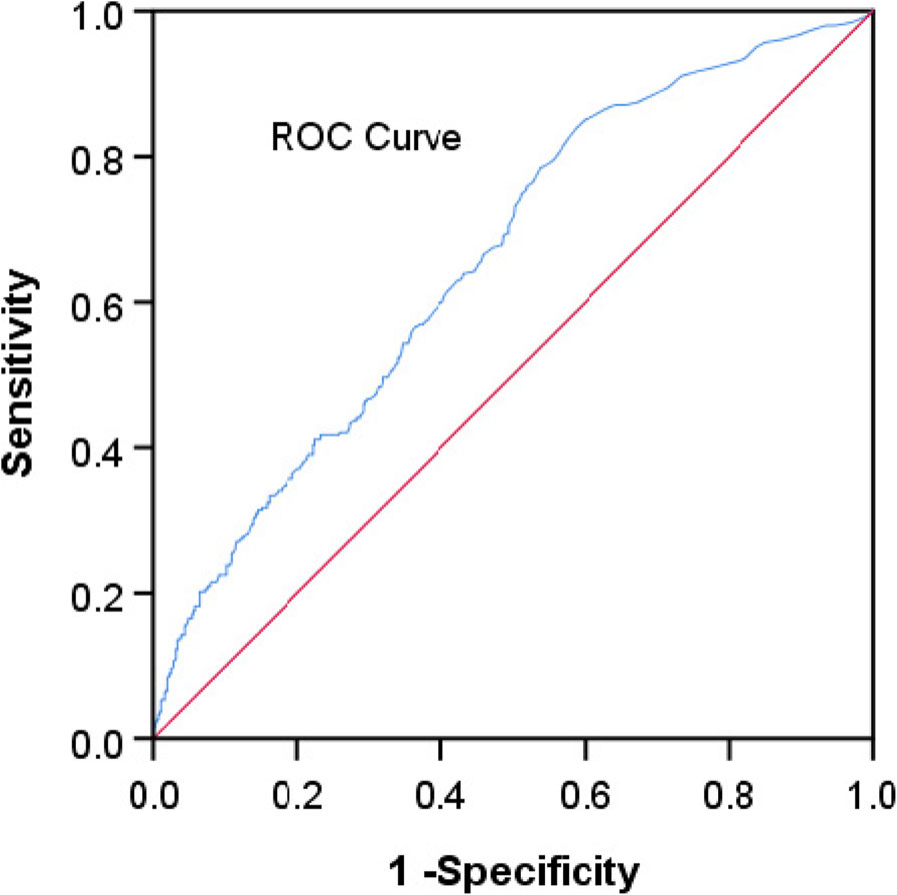
Receiver operating characteristic (ROC) curves were used to evaluate the predictive values of plasma D-dimer levels for poor outcome (area under the curve: 0.657; 95% CI, 0.620–0.694; *P* = 0.000)

To further evaluate the predictive values of plasma D-dimer levels in patients with AIS, the ROC curves and AUCs were created and are depicted in Fig. 4. Based on the ROC curve, the optimal cut-off value of plasma D-dimer levels as an indicator for diagnosis of unfavorable functional outcome was projected to be 0.315 mg/L, which yielded a sensitivity of 83.8% and a specificity of 41.4%, the AUC was 0.657 (95% CI, 0.620–0.694; *P* = 0.000).

## Discussion

In the present study, higher plasma D-dimer level on admission was a significant independent determinant of short-term neurological dysfunction in patients with AIS within 90 days in a Chinese population. After adjusting for various confounders, the correlation remained significant.

Previous prospective epidemiological investigations have concluded that there is a positive association between plasma D-dimer levels and stroke [26–28]. In some studies, the results showing that plasma D-dimer levels were associated with stroke severity [29, 30], infarct volume [15, 31, 32], and progression of stroke status [14, 33, 34]. However, the relationship between plasma D-dimer levels and functional outcome in patients with AIS has been poorly studied.

The available studies on stroke have shown relationships between plasma D-dimer level and functional outcome in several different population types with AIS [35–39]. Nam et al. [35] and Nezu et al. [36] found a predictive role of plasma D-dimer levels only in patients with cryptogenic stroke. A Canadian study by Kim et al. [37] reported the prognostic value of plasma D-dimer level in patients with noncardioembolic stroke. In a study of a Chinese population with complicating coronary heart disease, the result indicated that higher plasma D-dimer levels had a worse outcome within 90 days after the initial onset of AIS [38]. A Swiss study by Hsu et al. reported that a high plasma D-dimer levels indicates an unfavorable outcome in patients with AIS receiving intravenous thrombolysis [39]. However, on reviewing previous literature, we also found that some other studies have reported conflicting results. A report by Squizzato et al. [19] revealed that plasma D-dimer level in patients with AIS probably does not predict the functional outcome after adjustment for age and stroke subtype. Furthermore, two other studies did not even find a meaningful association between plasma D-dimer levels and the prognosis of patients with AIS [40, 41].

In this study, because the prognostic value did not alter even after adjusting for various confounders such as age, sex, BMI, vascular risk factors, baseline NIHSS scores, and stroke etiology, our results revealed plasma D-dimer levels are an independent biological prognostic marker of AIS. In fact, the positive value of plasma D-dimer in patients with all subtypes of AIS was indicated in several previous studies [33, 41, 42], which is consistent with our findings.

D-dimer derived from the cross-linked fibrin network is a final soluble fibrin degradation product which undergoes plasmin-mediated degradation [13]. Plasma D-dimer could be elevated in a population with thrombotic diseases such as pulmonary embolism and venous thromboembolism [42, 43], however, the mechanism remains unclear. There are several possible explanations for why plasma D-dimer levels might be relevant to poor functional outcome in patients with AIS. For instance, plasma D-dimer level increases in blood coagulation and degradation of fibrin and could be a marker of thrombosis based on the underlying mechanisms [44, 45]. Moreover, a high plasma D-dimer levels may result in resistance to the endogenous fibrinolytic system and influence thromboembolism formation [40, 46]. Furthermore, plasma D-dimer also stimulates the immune system and leads to changes in inflammatory mediators levels such as IL-1, TNF-alpha, IL-6, and IL-8 [47, 48]. Activated inflammation may contribute to the pathological alteration in patients with AIS [49]. In addition, infarct volume, initial stroke severity, and progression of stroke status were correlated with a high plasma D-dimer levels [14, 29–34], therefore elevated plasma D-dimer levels may predict poor outcome through the aggravation of cerebral tissue damage by disturbing recanalization and increasing reperfusion injury. Additionally, the plasma D-dimer levels in patients with AIS may identify those who may benefit from additional interventions, targeting some of the mechanisms mentioned above. This needs to be explored in further studies.

The present study has several limitations. First, this is a single-center, observational study. The sample sizes of patients are small, and selection bias was a major concern, thereby limiting the power to generalize our results. Second, the plasma D-dimer levels were measured only in the morning after at least 8 h of fasting in our study, however, recording the serial change of plasma D-dimer levels might better explore the correlation between D-dimer and outcome after AIS. Finally, our study explored the short-term outcome with an end-point defined at 90 days. The correlation between plasma D-dimer levels and long-term prognosis requires further confirmation in our study population. Therefore, further multicenter studies with a larger sample size need to be conducted.

## Conclusions

Elevated plasma D-dimer levels on admission are significantly associated with poor outcome after admission for AIS, suggesting a high plasma D-dimer level within 72 h of a stroke as a predictive marker for short-term poor outcome after 90 days in patients with AIS. Plasma D-dimer level is a convenient and economical biological indicator that could be used for improving the specific management of stroke rehabilitation and functional outcome.

## Data Availability

The datasets used and/or analyzed during the current study are available from the corresponding author on reasonable request.

## Abbreviations

AIS: Acute ischemic stroke
ANOVA: Analysis of variance
AUC: Area under the ROC curve
BMI: Body mass index
CAD: Coronary artery disease
CI: Confidence interval
DBP: Diastolic blood pressure
FPG: Fasting plasma glucose
IL: Interleukin
IQR: Interquartile ranges
mRS: Modified Rankin scale
NIHSS: National Institutes of Health Stroke Scale
OR: Odds ratio
ROC: Receiver operating characteristic
SBP: Systolic blood pressure
SD: Standard deviations
TNF: Tumor necrosis factor
TOAST: Trial of Org 10,172 in Acute Stroke Treatment
